# Smart Nanoformulation Based on Polymeric Magnetic Nanoparticles and Vincristine Drug: A Novel Therapy for Apoptotic Gene Expression in Tumors

**DOI:** 10.3390/life11010071

**Published:** 2021-01-19

**Authors:** Sharafaldin Al-Musawi, Sumayah Ibraheem, Salih Abdul Mahdi, Salim Albukhaty, Adawiya J. Haider, Afraa Ali Kadhim, Kadhim Ali Kadhim, Haitham Ali Kadhim, Hassan Al-Karagoly

**Affiliations:** 1 Faculty of Biotechnology, Al-Qasim Green University, Babylon 51013, Iraq; drsalih@biotech.uoqasim.edu.iq; 2 Al_kindy College of Medicine, University of Baghdad, Baghdad 10071, Iraq; sumayahibraheem@kmc.uobaghdad.edu.iq; 3 Department of Chemistry, College of Science, University of Misan, Maysan 62001, Iraq; albukhaty.salim@uomisan.edu.iq; 4 Applied Science Department/Laser Science and Technology Branch, University of Technology, Baghdad 10066, Iraq; 100081@uotechnology.edu.iq; 5 Department of Biology, College of Science, Mustansiriyah University, Baghdad 14022, Iraq; afra.alaskaree@uomustansiriyah.edu.iq; 6 Department of Pharmacy, Al-Yarmouk University College, Baghdad 56001, Iraq; Pharma.drkaka@al-yarmok.edu.iq; 7 Iraq Ministry of Health, Medico Legal Directorate, Baghdad 10011, Iraq; Medhaith07@yahoo.com; 8 Department of Internal and Preventive Medicine, Veterinary Medicine College, University of Al-Qadisiyah, Diwaniyah 58002, Iraq; hassan.aliwee@qu.edu.iq

**Keywords:** nanocomposite, vincristine, apoptosis, gene regulation, testicular tumor cells

## Abstract

Background: Advanced nanobiotechnology provides safe and efficient drug delivery systems to deliver chemotherapy that targets cancer cells efficiently. Methods: A polymeric-magnetic nanocarrier was composed of a dextran (DEX) shell, a superparamagnetic iron oxide (SPION) core and was conjugated with folate (FA) to carry the anticancer drug vincristine (VNC) in Tera-1 testicular tumor cells. The molecular mechanisms by which apoptosis was induced were analyzed using flow cytometry and qPCR, which exhibited anticancer activity of nanoparticles (NPs). Results: This nanocarrier revealed a controlled release of VNC in citrate and phosphate buffer solutions that were maintained at pH 5.5 and pH 7.4, respectively. The Inhibitory concentration (IC50) values were greater than 5 mg/mL and displayed ten times higher cytotoxicity than the comparable free drug concentration. The Caspase-9 and P53 expressions were increased, whereas P21 and AKt1 decreased noticeably in the treated cells. The results point to the possible activation of apoptosis following treatment with NPs loaded with vincristine.

## 1. Introduction

**Scheme 1:**
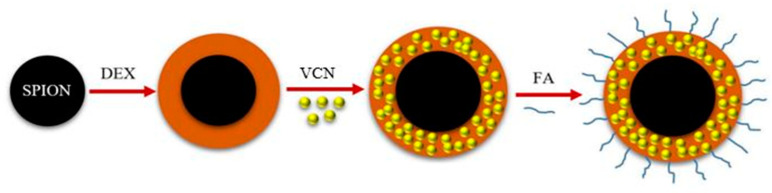
Schematic presentation of the method for preparation and synthesize FA-DEX-VNC-SPION nanoparticles. Abbreviation: FA folate, DEX, dextran, VNC, vincristine, SPION, superparamagnetic iron oxide nanoparticles.

Vincristine (VNC) is a strong anticancer drug that belongs to a group of drugs called vinca alkaloids [1]. The antitumor mechanism of the VNC drug is known for stopping the cancer cells from dividing. Therefore, it inhibits the cancer progression and metastasis [2]. VNC, which is also known as leurocristine and is sold under the Oncovin branded product. It is an anticancer medication typically combined with other medications to treat different forms of tumors including leukemia, Hodgkin’s disease, brain and lung carcinoma [3,4,5,6]. However, the main challenge for VNC is its cytotoxicity and nonspecific bio-distribution, which cause severe side effects [7]. Through inserting the active ingredient into suitable nanomaterials with ideal size, surface and charge characteristics [8,9], the drug covalently attached SPION has been studied in order to provide tumor-site anticancer agents [10]. Researchers have drawn considerable attention to specific features of SPIONs, like nontoxicity and fast external magnetic field reactions, especially in the identification and treatment of potential cancers. In addition, several biocompatible polymers including poly-l-lysine (PLL), chitosan, dextran, and polyethylene-glycol (PEG) have been used to develop SPIONs in drug and gene delivery systems. Dextran has been extensively and efficiently used mostly for various biomedical purposes. The DEX encapsulation of SPIONs provides desirable stability with no toxicity reported [11,12,13]. Besides that, an efficient nanostructured framework is provided for potential treatment delivery to the target [14]. These systems enable local medication to be administered extensively and provide an improvement in medication concentrations within cancer cells, including moderate and minimum bloodstream concentrations of the drug [15]. The surfaces of different tumor types, such as testicular cancer cells, were overexpressed with folate (FA). It was also used to target treatment agents for cancer [16]. This study established a stimulus-responsive regulated VNC drug release with biocompatible, tumor-specific properties based on the FA-DEX-SPION nanoscale formulation to combine those shown in Scheme 1 for testicular cancer cells. In addition, by corroborating selected findings of the RT-PCR study, the gene expression levels were extended.

## 2. Materials and Methods

### 2.1. Materials

All materials used in this study such as VNC sulfate, dextran, DMSO, FeCl_3_·6H_2_O, FeCl_2_·4H_2_O, MTT, NH_4_OH, and Folate were purchased from Merck company. Tera-1 and Hs1 cell lines were purchased from ATTCC, UAS.

### 2.2. Manufacture of DEX Coated SPION Nanoparticles

The SPION was manufactured using the co-precipitation technique, as previously mentioned, but with some modifications [15]. N_2_ gas was transferred to 58 mL of double distilled water. In the suspension, FeCl_2_·4H_2_O (two mmol), mmol of FeCl_3_·6H_2_O (four mmol) and 0.6 % dextran (ten mL) were then injected, and NH4OH was added dropwise with a syringe, under a nitrogen atmosphere. The formulations were stirred at 65 °C for 30 min, and the black precipitates were obtained.

### 2.3. VNC-Loaded Nanoparticles

25 mg of the VNC drug (initially diluted by 20 mL of DMSO) was added to the modified DEX coated SPION (120 mg), and mixed for nearly one day. FA was then added to the mixture and incubated by shaker incubator (200 rpm) at room temperature for 1.5 h, resulting in obtaining FA-DEX-VNC-SPION. The FA-DEX-VNC-SPION was separated at 15,000 rpm through centrifugation and then washed with distilled water three times. By applying UV-Vis spectroscopy at a 425 nm wavelength, the unloaded VNC was measured by calculating its supernatant concentration.

(1) $$Encapsulation\;efficiency\;\left(\%\right)=\frac{\left(Total\;Q\;of\;M-free\;Q\;ofM\right)}{Total\;amount\;of\;M}\;\times \;100$$

### 2.4. Characterization

TEM and SEM were used to estimate the size and morphology of FA-DEX-VNC-SPION. A small volume of the solution was placed on a cover slide that allowed it to dry until visualization at room temperature. Zeta potential and DLS (MALVERN, Nano S, London, UK) were achieved after suspended in distilled water and diluted to an acceptable concentration. In an FTIR device (Shimadzu Company, Tokyo, Japan) the formulated nanosystems were evaluated over a 250–4500 cm^−1^ wavelength area to describe the functional groups in the samples. Additionally, to test the magnetic properties, VSM Lakeshore 7404, Westerville, OH, USA) was carried out.

### 2.5. Drug Release Measurements

The release of VNC drugs from the nanocomposite was carried out in buffers of phosphate (PBS) (0.01 M with pH = 7.4), and citrate (0.01 M with pH = 5.4) at 37 °C. Two separate dialysis bags were poured with 1 ml of the drug-loaded micellar solution. These bags were set in (100 mL, 0.01 M) citrate buffer and phosphate buffer. To prevent the potential precipitation of the released VNC, Tween 80 as the emulsifier was applied to each of these buffers. The temperature was set at 37 °C, and a shaker (GFL, Burgwedel, Germany) was used to agitate the buffer gently. Sampling was conducted at different time intervals. 200 μL of the sample was taken at each point of time for freeze-drying. The remainder was dissolved in 2 mL of methanol. By nano-dropping, the quantity of the released VNC was calculated. The release of the drug was calculated using the following

(2) $$R=\frac{V\;\sum_{i}^{n-1}C_{i}+V_{o}C_{n}}{m_{\mathrm{drug}}}$$

R is the release of drug (percent), the sampling volume indexed by V, V_0_ is the initial drug volume, the drug concentrations were presented using Ci and Cn, “i” is the testing moment as well as “n”, and the encapsulated drug mass in nanocarrier is showed by m_drug_. The sedimented substance was washed with double distilled water again and suspended.

### 2.6. Cell Culture Condition

Tera-1 and Hs1 cells were incubated in culture media (DMEM), augmented with fetal bovine serum (FBS), penicillin/streptomycin, and cultured in 5% CO_2_ in an incubator at 37 °C.

### 2.7. Cellular Internalization

Fluorescein 5(6)-Isothiocyanate (FITC) functionalization of VNC loaded FA-DEX-SPION was performed to verify the efficiency of its cell internalization using fluorescence microscopy. 10 μg of FITC functionalized nanodrug system was applied for the incubation of the cells at three hours. The NPs-included media was subsequently expelled and washed repeatedly with phosphate-buffered saline (PBS). To perform cell photography, a fluorescence microscope was used.

### 2.8. Cytotoxicity Assay

A 96-well cell plate was applied for cytotoxicity study. 200 μL of medium containing 1 × 10^4^ cells was utilized for adding into each well and allowing the cells to grown for 24 h. The culture medium was removed from all wells and substituted with a new one containing different concentrations (10–60 μM), either drug, drug-loaded nanocarrier or nanocarrier, for 24 and 48 h incubation periods. The control was a group of cells without treatment. 10 μL of 5 mg/ml MTT solution per 100 μL media was added into each well. All plates were placed in an incubator at 37 °C and 5% CO_2_ for four hours. The residual MTT solution was removed, and 100 μL of DMSO was added to each well to dissolve the formazan crystals. To ensure that the crystals of formazan were dissolved properly, a vibrating plate was used to shake the plates for five minutes, with a multi-scan plate reader, estimating the absorption of each well at 540 nm. The outcomes were obtained using mean ± SD.

(3) Relative cell toxicity=[(Asample−Acontrol)/Acontrol] × 100

### 2.9. Apoptosis Estimation

A flow cytometry technique was used to measure the total apoptosis and necrosis in cells subjected to various VNC-loaded FA-DEX-SPION treatments, VNC, and folated DEX-SPION at 48 h with approximately 10^4^ cells/well. All cells were detached by trypsin enzyme and, after counting, placed in each well of six-well plates. Propidium iodide (PI) and Annexin V–FITC were using to stain the cells. Based on the manufacturer’s protocols, Apoptosis was evaluated by Annexin V-FITC apoptosis detection kit.

### 2.10. RT-PCR

The total RNA was isolated from Tera-1 cells using TRIzol (Invitrogen Life Technologies, UK) after 48 h treatment with drug loaded nanocarrier, void drug and bare nanocarrier. The RNA concentration was quantified by calculating the optical density at 260/280 wavelength. cDNA synthesis from the total RNA was performed using cDNA synthesis (Fermentas, Germany). As shown in Table 1 reverse and forward primer for β-Actin, P53, P-21, Caspase-9 and Akt-1 genes were synthesized and utilized in this work.

To evaluate the gene expression of P53, P-21, Caspase-9 and Akt-1, real-time PCR was achieved. Beta-actin was chosen as a reference gene. 10 μL of SYBR Green, 5 μL of cDNA and 0.5 μL of each particular primer were used in the amplification reactions. PCR was carried out for 50 cycles (the temperature, time and number of cycles for each step are mentioned in Table 2). RT-PCR success was then examined by melting curve analysis.

## 3. Results and Discussion

### 3.1. Nanoparticles Characterization

**Figure 1:**
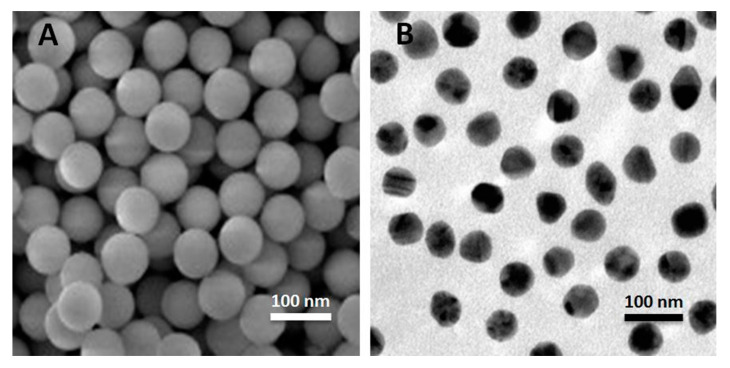
Microscopic analysis of FA-DEX-SPION NPs. (**A**) SEM image and (**B**) TEM image.

**Figure 2:**
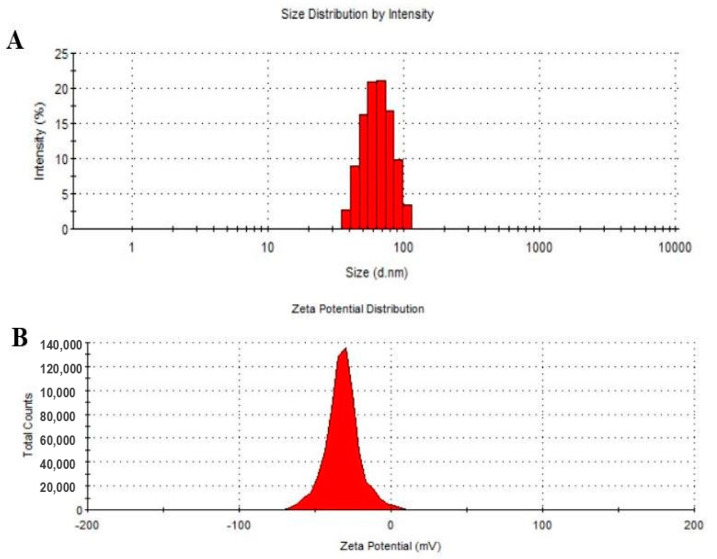
Images of FA-DEX-VNC-SPION nanoformulation size (**A**) and charge (**B**) using DLS. (*n* = 3).

To evaluate the form and surface characteristics of the prepared NPs, SEM and TEM were employed. The SEM and TEM images show that synthesized NPs, possessed a spherical form with a clear surface and remarkable dispersity ratio (Figure 1). The size and distribution of NPs were computed using dynamic light scattering (DLS) (Malvern Zetasizer ZS, Malvern, UK). This indicated that the NPs size and zeta potential were ~74 ± 12 nm and 43 mV, respectively (Figure 2A,B). Particle size is one of the most important parameters for controlling the nanoparticles biocompatible and bio-active properties. The size of the particle is also a crucial factor as it has a close association with the formulation of stable nanocarrier [17]. Earlier, N. Kumar et al. [14] observed spherical shaped vincristine-loaded folic acid-chitosan conjugated nanoparticles with a granule like structure due to loading of vincristine.

**Figure 3:**
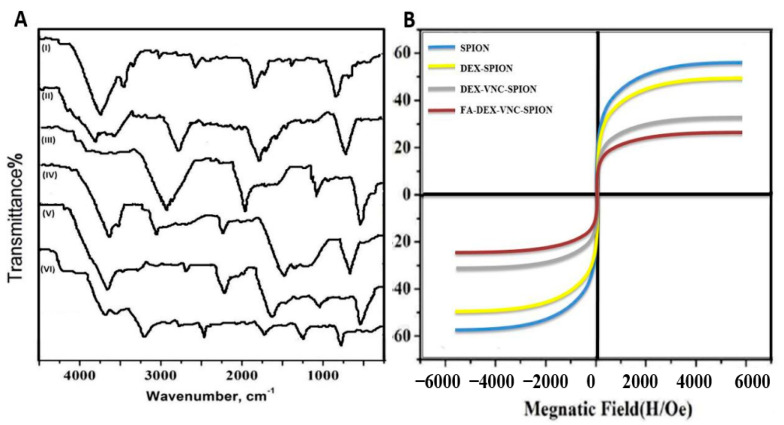
FTIR study of (I) DEX, (II) SPION, (III) VNC, (IV) FA, (V) DEX-SPION and (VI) FA-DEX-VNC-SPION (**A**), The magnetization study of SPION, DEX-SPION, DEX-VNC-SPION and FA-DEX-VNC-SPION at 400 K (**B**).

An FTIR test was applied to check the surface functional groups of the synthesized nanoformulation with 3300 and 1600 cm^−1^ peak DEX absorption spectra, respectively, as shown in Figure 3 (I). The existence of high SPION absorption peaks appeared at approximately 465.5 cm^−1^ and 571.3 cm^−1^ Figure 3 (II). The 591 cm^−1^ band was proved as the Fe-O stretching vibration of the tetrahedral sites of the spinal structure [18]. The free FA (stretching vibration of the benzene ring skeleton at 1495/cm^−1^) infrared spectrum max is shown in Figure 3 (IV). Distinct peaks of absorption of approximately 2920 cm^−1^ (overlapping vibrations of C-H stretching methyl, methylene and-CH) and 1690 cm^−1^ (stretching vibration peak of group C=O) are indicated in Figure 3 (III), which indicates the loading within FA-DEX-SPION NPs of the VNC drug [18].

Vibrating sample magnetometry (VSM) (b) was used to define the magnetic properties of NPs, as shown in the unmodified form of SPION, DEX-SPION, VNC loaded DEX-SPION and VNC loaded FA-DEX-SPION. The VSM of the unmodified SPION was 57 emu/g, compared to 41 emu/g for the DEX-coated SPION, and 37 and 30 for the VNC loaded DEX-SPION and VNC loaded FA-DEX-SPION, respectively. The presence of a notable amount of diamagnetic dextran in the nanoformulation was responsible for this decrease in saturation magnetization [19].

### 3.2. Drug Release Study

**Figure 4:**
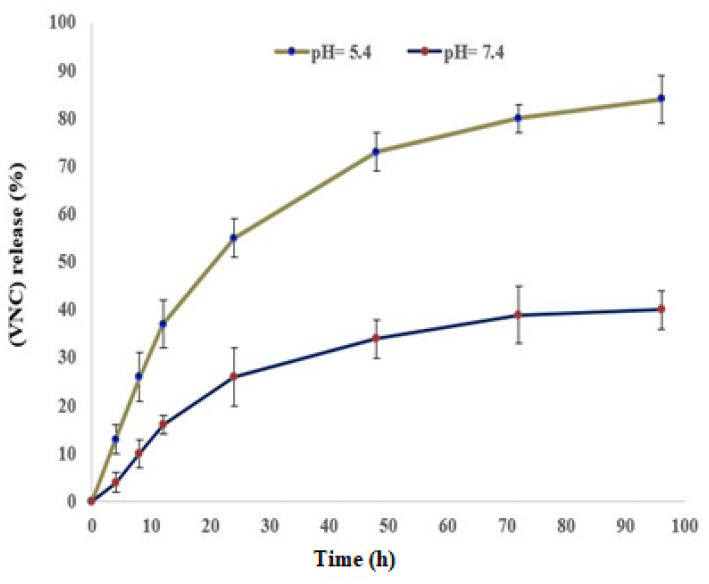
Drug release curve of nano-formulation with VNC loaded FA-DEX-SPION at different pH (7.4 and 5.4) and 37 °C. The mean values ± SD (*n* = 3) are defined by the data.

The volume of VNC drug released from its nanocarrier is presented Figure 4. The loading level showed VNC rapid adsorption and, after 90 min, the adsorption rate slowed down. This is because the surface of the nanoparticles was filled with VNC [20]. Also, the core-shell structure may affect this relation. Additionally, the polymer structures supply many functional groups for several notable interactions with the VNC drug structure on the surface of the nanosystem [21]. Centered on the outcome of the curves of in vitro releases shown in Figure 4, the VNC releases rate from its nanocarrier over 96 h and, under the same conditions, was faster in the acidic pH (5.4) of the citrate buffer compared with phosphate buffer with a standard pH of 7.4. The stability of FA-DEX-VNC-SPION in buffers and different pH solutions was checked using DLS analysis to identify the change in FA-DEX-VNC-SPION stability. The data proved that FA-DEX-VNC-SPION retained an approximately similar size (74 ± 14) and dispersity without any aggregation. Al-Musawi et al. showed that pH has an effect on controlling the encapsulation efficiency (%) and loading capacity (%) of carrier system for an anticancer drug [15,16].

### 3.3. VNC Drug Internalization of Cells

**Figure 5:**
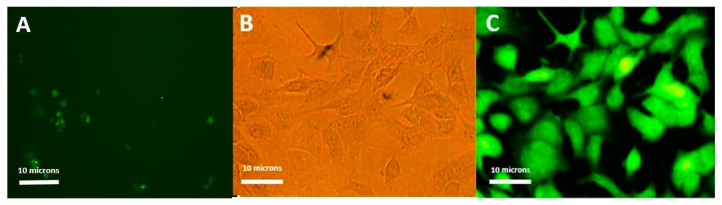
Cell internalization analysis of FA-DEX-VNC-SPION utilizing fluorescence microscopy in Tera-1 cancer cell (400× magnification). Image of VNC-treated cells with fluorescence microscopy (**A**). FA-DEX-VNC-SPION-treated cells (**B**) optical microscopy image. Fluorescence microscopy image of FA-DEX-VNC-SPION-treated cells (**C**).

In the Tera-1 cancer cells, the FA-DEX-VNC-SPION system was effectively internalized and was clearly analyzed by fluorescence microscopy (Figure 5B,C), while VNC alone was grouped as crystals bodies of different sizes (Figure 5A). The Tera-1 cells were treated with VNC-loaded nanocarriers and were bright green color due to the large internalization capacity. This property resulted from the enhancing solubility level of VNC after loading into the nanocarrier. Green, star-like, and insoluble fragments were observable in cells treated with VCN alone due to their insolubility in an aqueous medium. This finding clearly demonstrates that FA-DEX-VNC-SPION is a really powerful carrier that can be used for Tera-1 cancer cell-targeted drugs as a delivery mechanism. The results were considered satisfactory for targeted applications and are in agreement with those previously published [9,11,13,15].

### 3.4. The MTT Assay

**Figure 6:**
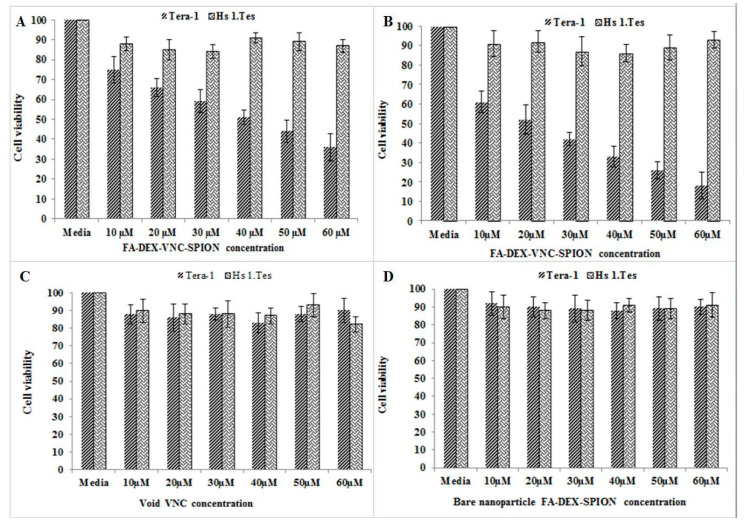
Cytotoxic effect of various concentrations (10–60 μM) of treated FA-DEX-VNC-SPION after 24 h (**A**), 48 h (**B**) and free VNC (**C**) & FA-DEX-SPION (**D**) at 48 h on both Tera-1 and Hs1 cell lines. (*n* = 3).

The biocompatibility of the nanocomposite was assessed using the MTT test through incubation of Tera-1 and Hs1cell lines with the prepared FA-DEX-SPION NPs-loaded VNC (10–60 μM) for 24 h. Along with a high concentration of FA-VNC-DEX-SPION NPs (60 μM), the findings showed high viability (>80 percent). Figure 6 demonstrates that FA-DEX-SPION-embedded VNC NPs are possible biocompatible materials to deliver VNC with sufficient stability. Similarly, Albukhaty et al. [22] determined the same cytotoxicity effect of free vinblastine drug and its nanoformulated form in same nanoparticle (FA-DEX-SPION).

### 3.5. Apoptosis Measurement by Flow Cytometry

**Figure 7:**
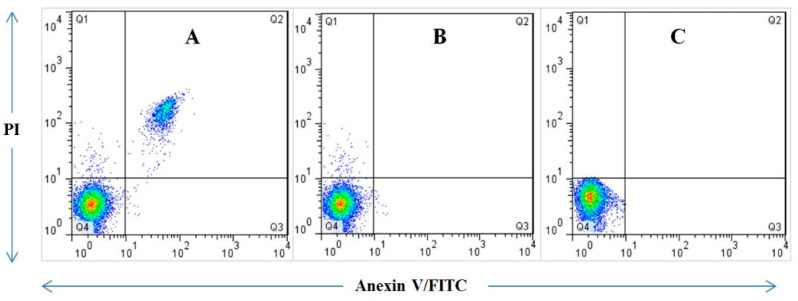
Induction of Apoptosis by VNC drug-loaded nanocarrier. The Tera-1 cell line is treated with (**A**) VNC drug-loaded nanocarrier (**B**) bare nanocarrier and (**C**) free VNC drug. The apoptotic Tera-1 cells increase when treated with VNC drug loaded nanocarrier. (*n* = 3).

After treatment with both the free VNC, FA-DEX-SPION and VNC loaded with FA-DEX-SPION formulation for 48 h, Annexin V and PI were utilized for staining the Tera-1 cells for apoptosis analysis. Cells without treatment were used as a control group. Results showed that the nanodrug system is appropriate to use for VNC-delivery. Furthermore, the apoptotic effects of the FA-DEX-VNC-SPION were examined with a higher apoptosis rate in comparison to the free VNC or FA-DEX-SPION alone (Figure 7). The percent of apoptosis for treated cells was identified. In addition, treatment of the Tera-1 cell line separately with bare nanocarrier and free VNC drug suggested that both treatments did not demonstrate a notable apoptotic stimulation. The apoptotic stimulation was obtained utilizing a similar nanocarrier concentration. More distinctly, the FA-DEX-VNC-SPION led to 42% apoptosis, 39% late apoptosis (Q2 square) and 3% early apoptosis (Q3 square) respectively but the apoptotic induction rate of free VNC and void FA-DEX-SPION nanoparticles on the Tera-1 cancer cell line was insignificant. Apoptosis can occur in both physiological and pathological conditions as an orchestrated cellular mechanism [22]. Cell proliferation is out of control in cancers, and apoptosis is inhibited [23]. When cell cycle arrest happens, cell proliferation is reduced. In an attempt to repair the damage, cell cycle arrest will be triggered if DNA damage occurs. However, the cell will undergo cell death in an apoptosis manner if the damage is too severe to be reversed [24].

### 3.6. Gene Expression

**Figure 8:**
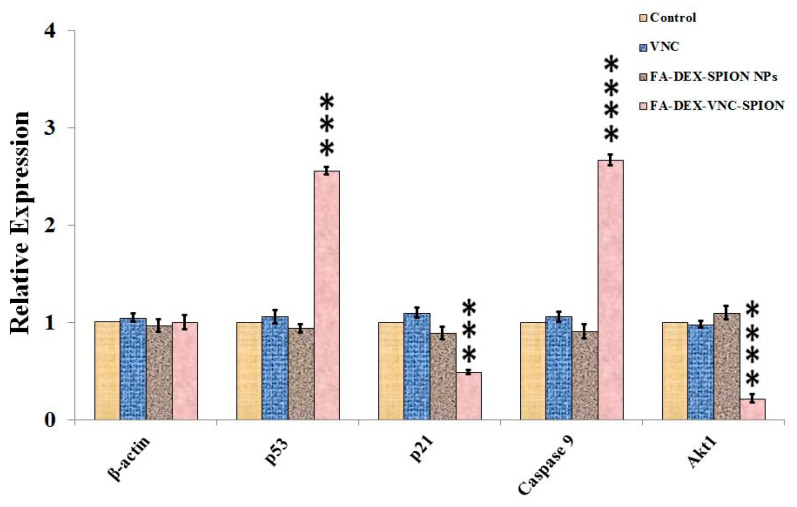
The gene expression study using RT-PCR. Tera-1 cell lines treated with free drug, nanodrug system, and bare nanocarrier without drug. The curve values showed mean ± SD (*n* = 3). **** *p* < 0.0001, *** *p* < 0.001, and represent remarkable variation between the control (without treating) and other mentioned treatments.

RT-PCR tests showed the up-regulation of P53, P21, Caspase-9 and AKT-1 expressions in FA-VNC-DEX-SPION Tera-1-treated cells, which indicates the significance of these selected genes in the current apoptotic phase. The expression values of the P53, P21, Caspase-9 and AKT-1 genes were investigated using RT-PCR. The beta-actin gene was selected to be the reference control gene. All genes had noticeable differences between malignant and nonmalignant samples in terms of expression, as shown in (Figure 8) Beta-actin expression levels stayed unchanged between the control and the cancer cells, either with or without VNC therapy. Besides that, the level of expression of the Caspase-9 was high in cancer cells (**** *p* < 0.0001) when treated with VNC drug-loaded nanocarrier compared to free VNC drug and bare nanocarrier. Previous studies confirmed that P53 and P21 act as protein tumor suppressors [25]. The results indicated that P53 is expressed at high levels in the treated Tera-1 cells by VNC drug-loaded nanocarrier, as demonstrated by RT-PCR. Effective targeting was transported to the action site through interacting with its receptor on the cellular membrane, which suggested its inhibition properties on the proliferation and growth of cell cancer cells. On the other hand, P21 and AKT-1 genes played a key role in tumor induction, and their lowest expression level in cells presented good indicator for therapeutic effect [26,27,28]. Moreover, low expression levels of the P53 and Caspase-9 genes in cancer cells were remarkably decreased when treated with VNC drug-loaded nanocarrier compared to VNC and bare nanocarrier. This indicates that the medication was effectively transferred to the cancer cells’ target site.

## 4. Conclusions

The DEX-SPIONs conjugated by the FA-carrying chemotherapeutic agent VNC were successfully prepared using the co-precipitation technique. With the nano-size distribution of particles in Tera-1 cells, the nano vehicle enhanced the encapsulated drug. The established FA-DEX-VNC-SPION had a sustainable drug release, leading to targeted testicular cell-toxicity that was dose- and time-dependent. FA-DEX-VNC-SPION had an inhibiting activity against tumor growth in the TERA-1 cancer cells, which was more marked than VNC and FA-DEX-SPION by themselves. Moreover, the high biocompatibility, loading efficiency, controllability and penetrability of the FA-DEX-VNC-SPION made it a valuable and interesting method for a broad range of possible nanomedicines. It is suggested that FA-DEX-VNC-SPION can stimulate cytotoxic activity with gene expression levels of P53, P21, Caspase-9 and AKT-1, as well as a decrease in cancer cells. This would effectively control progression to void VNC and FA-DEX-SPION without toxicity to healthy cells. It could, therefore, be used as a safe and effective antitumor agent that could be used in clinical environments.

## Figures and Tables

**Table 1:** The primers for β-Actin, P53, P-21, Caspase-9 and Akt-1 genes in the present study.

Primer Name	Forward Primer (5′ 3′)	Reverse Primer (5′ 3′)	References
β-actin	CTGGCACCCAGCACAATG	GCCGATCCACACGGAGTACT	[17]
P53	AGGTGACACTATAGAATA	GGGATATCACTCAGCATG	[18]
P-21	AGGTGACACTATAGAATA	GGGATATCACTCAGCATG	[19]
Caspase-9	ACTTTCCCAGGTTTTGTTTCCT	GAAATTAAAGCAACCAGGCATC	[20]
Akt-1	AGGTGACACTATAGAATA	GTACGACTCACTATAGGG	[21]

**Table 2:** Temperature, time and number of cycles for each step.

Step	Temperature	Time	Cycles
Initial denaturation	95 °C	10 min	1
Denaturation	95 °C	15 s	48
Annealing	60 °C	1 min	
Melting curve analysis	95 °C	5 s/step	1

## Data Availability

Not applicable.
